# In reply to: “Post-COVID lung injury and the need for longitudinal histology”

**DOI:** 10.3325/cmj.2025.66.461

**Published:** 2025-12

**Authors:** Grgur Salai, Jasna Tekavec-Trkanjec

**Affiliations:** Department of Pulmonology, University Hospital Dubrava, Zagreb, Croatia; Both authors contributed equally

**In reply:** We thank Dr Khan for important remarks in their Letter to the Editor (1) regarding our previously published paper (2). In January 2025, we published a single-center cross-sectional study based on the histopathological analysis of transbronchial biopsies obtained from long-COVID patients during the COVID-19 pandemic. Our study found slow recovery of lung tissue with long-lasting diffuse alveolar damage (up to 9 weeks) and vascular abnormalities (up to 13 weeks) following infection onset (2).

Dr Khan raised important remarks and concerns, which we would like to systematically address:

First, the author stated that the cross-sectional design of our study makes it difficult to establish temporal progression. They highlight the need of obtaining combined serial radiologic and histopathologic data (1). We fully agree that the cross-sectional design is a major limitation in interpreting our analysis. However, due to ethical concerns, we decided not to systematically repeat transbronchial biopsies unless they were clinically indicated. We further agree that incorporating radiological and clinical parameters is vital for establishing diagnosis. Namely, all study participants were presented in our multidisciplinary team discussions (2).

Second, the author raised the point that the vast predominance of male participants may limit generalizability (1). We agree. Namely, as we included consecutive patients, our study was not designed to match patients based on sex or age. The cause of predominance of male participants in our cohort is not entirely clear. As previously hypothesized (2), male sex was associated with worse COVID-19 outcomes, which might explain the inclusion of more male participants in our study (3-6). Sex differences in histopathological (as well as clinical and radiological) findings in long-COVID patients need to be evaluated.

Third, the author further addressed the limitations of employing forceps biopsy, compared with cryobiopsies for obtaining lung parenchyma (1). We agree with the author. As we previously stated, cryobiopsy was not available in our center at the time of our study (2). We fully acknowledge that forceps biopsy samples may be more prone to sampling errors and interobserver variability than cryobiopsies (7,8). This major limitation was somewhat (although not entirely) offset by independent interpretations by two experienced pulmonary pathologists and a follow-up multidisciplinary discussion, which allowed for integration of clinical and radiological characteristics with the histopathological analysis (2).

Another important point was that the cases of lepidic adenocarcinoma and fibrosing non-specific interstitial pneumonia might have coexisted with COVID-19-related damage (1). We agree with the author that this is highly possible. Namely, unrecognized pre-existing respiratory conditions are often discovered during the post-COVID follow-up (6), so this might hold true (especially in the case of lepidic adenocarcinoma) (2). Since the causality can be neither established nor dismissed, these cases have to be reported along with other results. Additionally, pre-existing conditions, if present, might have been exacerbated by SARS-CoV-2 infection (9,10).

To conclude, we thank the author for taking an interest in our field of research and for making these important remarks. We fully acknowledge the limitations and potential sources of bias in our research. However, we deem that, in spite of these limitations, our work adds to the limited body of knowledge regarding *in vivo* lung tissue analysis of long-COVID patients.
